# Evolution and Translation of Research Findings: From Bench to Where?

**DOI:** 10.1371/journal.pctr.0010036

**Published:** 2006-11-17

**Authors:** John P. A Ioannidis

**Summary**

The credibility and replication of research findings evolve over time, as data accumulate. However, translation of postulated research promises to real-life biomedical applications is uncommon. In some fields of research, we may observe diminishing effects for the strength of research findings and rapid alternations of exaggerated claims and extreme contradictions—the “Proteus Phenomenon.” While these phenomena are probably more prominent in the basic sciences, similar manifestations have been documented even in clinical trials and they may undermine the credibility of clinical research. Significance-chasing bias may be in part responsible, but the greatest threat may come from the poor relevance and scientific rationale and thus low pre-study odds of success of research efforts. Given that we currently have too many research findings, often with low credibility, replication and rigorous evaluation become as important as or even more important than discovery. Credibility, replication, and translation are all desirable properties of research findings, but are only modestly correlated. In this essay, I discuss some of the evidence (or lack thereof) for the process of evolution and translation of research findings, with emphasis on the biomedical sciences.

## Translation of Basic and Preclinical Science

Translation of biomedical research findings to useful applications is a major challenge [1]. Thirty years ago, Comroe and Dripps [2] proposed that medical progress depends on basic research, but their methods and conclusions have been challenged [3,4]. Regardless, successful translation of research promises is uncommon. Among 101 articles published between 1979–1983 in six top basic science journals that clearly made promises for a major clinical application of their findings in therapeutic or preventive interventions, only 27 technologies were evaluated in a published randomized controlled trial (RCT) by 2003 [5]. Nineteen technologies were evaluated in at least one RCT with “positive” results, but only five of them are currently in licensed clinical use and only one is in wide clinical use today. Involvement of industry authors in the original basic science report and industry support increased translation to human experimentation 10- and 3-fold respectively.

Another study has examined [6] whether the results obtained in animal models of acute stroke guide the selection of agents for testing in humans. Across 1,026 agents tested in animals, the agents proceeding to human testing showed similar reductions in infarct size in animals as those that did not advance further. Thus selection for further translation did not seem to be guided by rational principles.

Other investigations have examined whether in vitro or in vivo biological research agrees with evidence on human participants on the same topic. One evaluation [7] of genetic polymorphisms showed no correlation between epidemiological odds ratios for disease susceptibility and in vitro effects on gene transcription in cell lines. Two other investigations addressed the concordance of epidemiological associations versus evolutionary conservation and tissue-based assays for genetic variants [8,9]. Despite some concordance, correlation was still modest.

The methodological quality of basic research is also largely understudied and there are only preliminary efforts to improve the reporting of basic and preclinical studies [10]. Rapidly evolving methods and technology are difficult to standardize. Nevertheless, animal studies with higher quality “scores” apparently find more precise and more conservative results than studies with lower “scores” [11,12]. Similarly, effect sizes appear larger in studies lacking randomization or blinding [13].

Some of the translation failure may be due to difficulties in communication between different fields in the spectrum of basic, preclinical, and applied research. Evidence-based medicine does not seem to have penetrated basic and preclinical science, while basic and preclinical research is often performed in a clinical and methodological vacuum (see Box 1).

## Diminishing Effects and the Proteus Phenomenon

Replication of research findings in different studies means that, allowing for random fluctuation in early investigations, accumulation of evidence from many studies should converge towards stable estimates that don't shift with additional data [14]. However, sometimes we see continuously diminishing effects over time. Even large effects, and prominent claims, may gradually disappear [15–17] as more data accumulate (Box 2) [18–21].

In the “Proteus phenomenon,” the first published study on a scientific question may find a most extravagant effect size; this is followed by the publication of another study that shows a large contradicting effect. Subsequent studies report effect sizes between these extremes [22]. Impressive findings have priority for publication. Strongly contradictory results may also have priority over replications and inconclusive results. The extent of this phenomenon across different disciplines needs more study.

**Box 1. Lack of Communication**

The *Journal of Biological Chemistry* is the premier biochemistry journal and the most cited journal across all sciences (the one receiving the highest number of citations). *Emerging Infectious Diseases* is the premier journal addressing new and rapidly evolving infectious threats that may have major repercussions for human health globally. The *Journal of Biological Chemistry* received 404,397 citations in 2005. Only nine of these citations were from *Emerging Infectious Diseases*—as compared with 38,676 from the *Journal of Biological Chemistry* itself and 9,272 from *Biochemistry-US.* Also, the *Journal of Biological Chemistry* made 237,572 citations in 2005. Only nine of these citations were to *Emerging Infectious Diseases*—as compared to 38,676 citations to the *Journal of Biological Chemistry* itself and 6,500 citations to *Cell.* If this seems like extreme isolation, it actually could be worse. In the year 2005, the *Journal of Biological Chemistry* never cited the *Journal of Clinical Epidemiology,* the premier journal on clinical epidemiology and research methods. Similarly, in the same year, the *Journal of Clinical Epidemiology* never cited the *Journal of Biological Chemistry.* [Data are derived from Thomson Scientific, Journal Citation Reports 2005.]

## Waves of Evidence Microcosms

New biomedical discoveries may try to cover widely the perceived knowledge gaps. This creates waves of new evidence microcosms. An example is shown in Box 3. However, some old microcosms are not abandoned, but continue their existence, supported by circles with their societies, meetings, and journals. Often they leave behind not only their few genuine discoveries, but also literature that may no longer be attractive to “outsiders,” even to contradict. While early refutations are attractive, kicking a dead horse is not. Documented refutation may then be less common than gradual fossilization.

Evidence microcosms may sometimes reflect true paradigm shifts [23]. However, new evidence microcosms may also arise simply because some new technology becomes available, not because scientists rationally perceive a crisis of accumulating anomalies in old evidence microcosms. These are not scientific revolutions, but simply searching under yet another new lamppost that happened to light up. Rational arguments may play a role in the diversion from one lamppost to another. However, the allure of grants, touting in prestigious journals and meetings, and plain novelty-seeking are also strong motives. Some lampposts may have few or no true discoveries to be made in their lit area. In these “null fields,” the claimed effect sizes of “discoveries” are simply accurate measures of the net bias operating in these microcosms.

## Are Clinical Trials Immune to these Problems?

RCTs are the most robust experimental design for studies involving humans. However, similar phenomena to those described above for basic/preclinical research may also apply to clinical trials research. Even *before* the advent of truly potent antiretroviral therapy, 25 RCTs identified interventions with statistically significant effects on survival of HIV-infected patients [24]; meta-analyses of published data suggested that approved, controversial, and contradicted interventions all shared similar effect sizes [25]. With current hindsight, several of the apparent survival benefits with these therapies (immuthiol, interferon, or immunoglobulin in adults, for example) seem non-credible. The HIV field experienced a wave of spuriously effective treatments before truly effective ones became available. In most clinical research, we have not witnessed yet the revolution now apparent in HIV therapeutics. Are some fields currently populated unawares by only seemingly effective interventions [26,27]?

Discrepancies and diminishing effects over time have been demonstrated even in fields where large trials are common, as with the use of nitrates and magnesium sulphate for acute myocardial infarction, for example [28]. In fields where small trials predominate, diminishing effects may be more likely [29,30]. As many interventions are introduced in a field, with most of them never compared head-to-head, indirect comparisons sometimes give incoherent conclusions [31,32]. Such inconsistencies pose questions about the internal [33] and external [34] validity of both the direct and indirect evidence.

Diminishing and refuted effects are more common in epidemiological than randomized research [35,36]. However, randomized evidence is not immune. The refuted claims that vitamin E and hormone replacement therapy may curtail cardiovascular mortality did not emerge only from the large Harvard cohorts, but also from equally highly cited trials with clinical or surrogate endpoints [37,38].

## Odds of Truth for Clinical Trials

The odds that a research finding will be true are small when effect sizes are small; when studies are small; when a field is “hot”; when there is strong interest in the results; when databases are large; and when analyses are more flexible [39]. To improve the credibility of research, one should increase pre-study odds, diminish bias, and enhance power.

**Box 2. Refuting a *Nature* Cover Page Story and a *p*-Value of 9 × 10^−16^**

In 1994, the cover page of *Nature* announced the discovery of the osteoporosis gene. A study of a few hundred subjects claimed that polymorphisms in the vitamin D receptor (*VDR*) gene could explain 75% of the genetic variability of bone mineral density [18]. This paper has received more than 1,000 citations to date. Three years later, the research team published an erratum in *Nature* acknowledging a laboratory error. The revised results showed a weaker, but still formally statistically significant association. Over 100 studies, mostly of small sample size, were performed trying to replicate this association. Several meta-analyses tried to synthesize the published data and concluded that the association was statistically significant. One of them [19] reached a *p* = 9 × 10^−16^ by comparing the proportion of studies with formally statistically significant results against an “expected” 5%— a questionable approach. A subsequent meta-analysis of individual level data from a small subset of studies also found an odds ratio of 4 for osteoporosis [20]. However, recent studies questioned the association. A very large study found a statistically significant association, but in the very opposite direction [21]. Finally, a large collaborative study with sample size about 100-fold larger than the original *Nature* study did not detect any association with either bone mineral density or fractures. All odds ratios in the main analyses were between 0.98 and 1.02, and not even 1% of the genetic variability of bone mineral density could be explained [52]. Nevertheless, this may not be the end of the story. *VDR* is a long gene with many haplotype blocks. In general, as the amount of data increases exponentially (e.g., genomic testing in biobanks with linkage to health outcomes), the potential for both discoveries and errors also increases exponentially.

Bias causes the proportion of statistically significant findings in the literature to be spuriously inflated. This *significance-chasing bias* includes publication bias, where the visible data are less than the real data; selective analysis and outcome bias, where the visible data are the real data, but presented or interpreted the wrong way; and fabrication bias, where the visible data are more than the real data. These biases may coexist in various combinations in a body of evidence.

**Figure 1:**
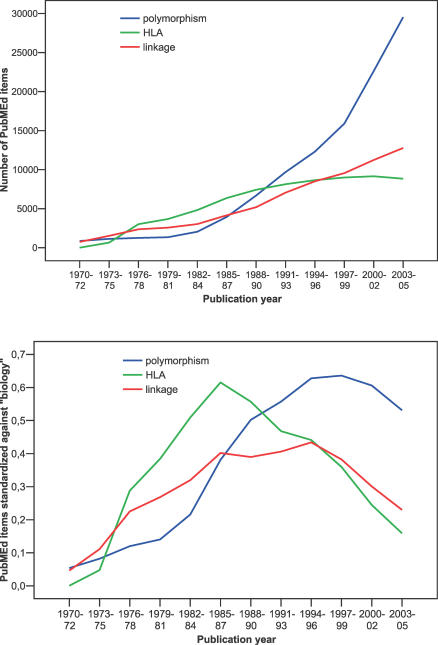
Three Waves of Evidence Microcosms in Genetics

In publication bias, studies with statistically significant results are more likely to be published than other studies. Trial registration [40] should diminish overt publication bias, but “negative” results may still be published later than “positive” results [41–43]. This time-lag may distort the literature for many years, enough time for a drug to carry its market share. With selective analysis and outcome bias, when investigators find “negative” results, they may change the outcome definitions or mode of analysis and thus find and publish results that cross thresholds of statistical significance. This bias is probably a greater problem than we thought, and very difficult to tackle [44–46]. Finally, fabrication bias is difficult to track, but fabricated data may arise even in the most visible clinical or other research.

**Box 3. Waves of Evidence Microcosms**

The search for genetic determinants of disease has been a fascinating field and it has witnessed shifts of attention in the last three decades: from human leukocyte antigens, to linkage studies with “whole genome” scans and testing of polymorphisms. Each wave has claimed thousands of relationships between genetic variation and human diseases. Some are confirmed, many are refuted, and probably even more are left behind in the literature, as new waves are created. These waves of evidence reflect wider waves of research in the life sciences. Figure 1 shows the results of simple PubMed searches for “HLA,” “linkage,” and “polymorphism.” All three show dynamic rises over time. However, one should also account for the general increase in the number of articles, in particular in the biological disciplines. The lower panel standardizes the number of PubMed items against the number of PubMed items for the term “biology” in the same time periods. The three waves peak in the mid-1980s, mid-1980s/mid-1990s, and mid-to-late 1990s, respectively, and decline thereafter, even if the total number of items continues to be high.

However, bias may be the least significant problem for RCTs. For randomized research, the main problem is probably the conduct of too many trials with poor scientific rationale and lack of clinical relevance; this translates to low pre-study odds. Most of these trials are also grossly underpowered. Evidence microcosms of randomized trials are built around themes (“lampposts”) where the incremental knowledge they can provide is minimal. Systematic reviews have found 254 randomized trials comparing different chemotherapy regimens in advanced non-small cell lung cancer [47]; 136 randomized trials comparing selective serotonin reuptake inhibitors against tricyclic/heterocyclic agents in depression [48]; and 666 articles on clinical trials of regimens for Helicobacter pylori [49]. Some clinical trials end up as tools for marketing, financing trial contractors, supplementing “clinical investigator” income, or creating petty CVs for promotion.

The current median sample size for RCTs is only 80 patients [50]. Even without any bias, if the pre-study odds are 1:10, a formally statistically significant (α = 0.05) finding from a small trial with 20% power has only 28% chance of being true.

**Box 4. Improving the Credibility, Replication, and Translation of Research Findings: Thoughts for Possible Solutions**

1. Promote multidisciplinary communication.
2. Foster systematic, evidence-based approaches to research.
3. Acknowledge in earnest the difficulty and even the failures of the scientific enterprise.
4. Examine which pathways have led to specific successes and failures in translation.
5. Focus on credibility rather than simply the statistical significance of research findings.
6. Synthesize evidence systematically from many studies and teams of investigators and anticipate this integration from the design phase of research.
7. Give credit to original ideas, good-quality work, and robust methodology rather than to impressive claims and magazine hype.
8. Encourage rigorous replication, not just discovery.

## Translation with Low Credibility

A seemingly effective intervention with low credibility may still be worth adopting, if it is safe—and affordable [51]. Otherwise we miss our small chances of benefit. One may also model the regret of accepting an intervention as effective while it is not [52]. However, besides uncertainty on benefits, we have even greater uncertainty about harms. The collection and reporting of information on harms of commonly used interventions and practices is deficient [53]. Surprises about late-discovered toxicities [54] may be only the tip of the iceberg.

Moreover, adoption of one scientific hypothesis may affect also our view of other hypotheses. With a domino effect, one research finding being accepted leads to other findings becoming seemingly more credible as well. This creates webs of information and practices to which we assign considerable credibility, while they may all be false and useless. Not surprisingly, this does not lead to successful translation.

## Increasing Credibility, Replication, and Translation

Evolution and translation of research findings does not have to be a roundtrip journey from bench to nowhere. In Box 4, I list some suggestions that may improve the situation. As we work on integrating scientific disciplines and materializing discoveries, translation would benefit from robust evidence. Translating non-credible, non-replicated research findings may have bleak consequences. We already have several useless prognostic and diagnostic tests, ineffective and possibly harmful therapies, and redundant sub-specialties sustained by unsubstantiated optimism on their benefits [55]. We should not add more junk to this pile.

As researchers, we should acknowledge difficulties and failures. In a world where everyone struggles to impress with achievements, public trust in science may be enhanced if it is seen as an enterprise where its workers do not simply try to impress, but seek the truth under often unfavorable odds of success. We also need to examine systematically what really has worked to date and the pathways of discovery for such successes. Moreover, we have a large evidence base where we can find out what has not worked so far and where and why we have been misled.

Research findings should be ascribed a credibility level that is different from their formal statistical significance. In the current era of massive hypothesis testing, levels of statistical significance are almost non-interpretable. The *p*-value threshold of 0.05, which barely worked when there were few hypotheses and investigators, is currently impractical. Circulating *p*-values increasingly reach depths of 10^−4^, 10^−10^, or 10^−60^. “Details” on how the data are collected, handled, and analyzed can change *p*-values by log scales.

In the past, we had few research findings; currently we have too many. This is exciting, but we don't know what they mean and how to use them. Credibility of research findings may be visualized in the form of a wide-based pyramid, where most findings have low credibility, and few have high credibility. RCTs can test findings that are somewhere between the middle to the top of the credibility pyramid. Target selection should be careful and systematically evidence based. Apart from attention to design, power, and protection from biases, this requires also careful strategic planning for designing research agendas and making sense of the overall picture of all RCTs in each field [56,57]. Designing trials in isolation or with non-scientific priorities creates fragmented, irrelevant evidence.

Finally, replication in the current era is probably as important as or even more important than discovery. Replication alone does not protect against bias. Studies with inherently bad design may be prone to replication if the same errors are repeated, while well-designed studies tend to replicate only when they are correct [58]. Replication requires rigorous evaluation with consistency in a variety of repeated tests. Scientific credit has traditionally been given to discoverers, but for many research fronts, discovery is currently an automated multiple testing process. The more difficult challenge is to dismiss false discoveries and materialize some truly useful findings.

## Abbreviations

RCT: randomized controlled trial
