# Correction: Evolution and Translation of Research Findings: From Bench to Where?

**DOI:** 10.1371/journal.pctr.0020003

**Published:** 2007-02-02

**Authors:** John P. A Ioannidis

In *PLoS Clinical Trials,* vol 1, issue 7: doi:10.1371/journal.pctr.0010036

In Box 2, Reference [52] should be corrected to [22]. References [18-21] in the text should be corrected to [18-22]. The six successive references from [22] to [27] in the text should be corrected to [23] to [28]. Finally, the current reference [28] in the text should be corrected to [14].

